# Treatment of Endometritis1Read before the Section on Obstetrics and Gynecology of the Buffalo Academy of Medicine, February 28, 1893.

**Published:** 1893-05

**Authors:** Herman E. Hayd

**Affiliations:** M. D., M. R. C. S., Eng., Buffalo, N. Y.; 78 Niagara Street


### TREATMENT OF ENDOMETRITIS.[1]

By HERMAN E. HAYD, M. D., M. R. C. S., Eng., Buffalo, N. Y.

Before accepting this paper, we must admit the existence of the disease called endometritis. There can be no doubt that the interior of the uterus is lined by a mucous membrane, and that it is clothed with epithelium, and has in its substance glands which secrete a material much like other mucous membranes. Like other organs of the body, it is the seat of congestions, catarrhs, and inflammations; and according as the intensity of this inflammation be greatest in the neck or body of this organ, so we have acute, subacute, and chronic cervical and corporeal endometritis. However, it must be understood that there cannot be any fast lines of limitation in these processes, as the one shades imperceptibly into the other. Moreover, we seldom see an inflammation confined absolutely to either neck or body of the uterus, but it extends by reason of continuity of tissue to the tubes, ovaries, and peritoneum, in varying degrees of intensity, and perhaps dependent upon the source of the infection, or at all events upon the cause and severity of the initial inflammatory onset.

But little need be said of the treatment of acute endometritis, since Nature rebels against local interference in these acute inflammations. Rest and quiet in bed, hot applications to the lower abdomen, codeia or morphia for the pain, and hot vaginal douches

1. Read before the Section on Obstetrics and Gynecology of the Buffalo Academy of Medicine, February 28, 1893.

given very gently, and only continued if grateful and comforting. At the onset, a brisk saline is not only good treatment for lessening the intensity of the disease, but it often cuts short and even aborts the attack.

If the cause be an acute specific infection, like gonorrhea, I am satisfied that dilatation and curettement under the strictest antiseptic precautions, and packing the uterine cavity with iodoform gauze, is a very brilliant and scientific procedure; because in these cases there is the greatest tendency to chronicity, and the possibility of irreparable mischief in the tubes and surrounding structures, even very early in the course of the disease. Frequent irrigation has its advocates in these acute septic and specific forms of endometrial trouble, by syringing the uterine cavity every hour or two with corrosive sublimate solutions; but that course of treatment which does away with the too frequent manipulation and handling of the parts gives the most satisfactory results; and, therefore, in my own practice, I prefer to make one operation, and with care and due cleanliness we can do no harm even in the most acute and virulent conditions. In the less acute inflammations, resolution is often brought about by properly applied glycerine tampons—either with boroglyceride, fifty per cent., or ichthyol, fifteen per cent., or glycerine and tannic—every other day, and on the intervening day to paint the vault of the vagina and cervix with Churchill's tincture of iodine, or to apply negative galvanism with Goelet's clay electrode, or intra-uterine galvanic applications, as advocated by me in previous papers on this subject.

The great surgical principle of drainage is nowhere in the whole domain of surgery more necessary for the proper management of the chronic and very intractable forms of the disease. An inflammation has extended from the mucous membrane into its glandular elements, structural disorganization, or excessive hypertrophy of them has taken place, the walls of the uterus, its connective tissue, and muscle fibre have been multiplied and developed, and, perhaps, the tubes, ovaries, and peritoneum seriously involved. The uterus has sagged downwards, and backwards, or forwards, and the escape of the profuse and constantly accumulating discharge is obstructed.

Now comes the consideration of the most difficult and perplexing problem in uterine and pelvic surgery. Is the endometritis, which has been responsible for the existing salpingitis, ovaritis, and peritonitis, the point of surgical attack, or has it ceased to be an important factor when the tube and ovarian mischief can, with reasonable certainty, be felt and diagnosticated? Naturally, the more the source of irritation be endometrial the more can we expect from local and conservative treatment, while, on the contrary, if tubal and ovarian disease exist to any greater degree, ablation of the offending organs is, perhaps, the only hope of relief. As a result of these premises, two great schools of pelvic surgery have their existence, the one believing that endometritis seldom requires any special treatment other than good hygiene, absence of sexual relation, good food, and good environment, tonics, and daily soluble movements from the bowels. In other words, ill-health and much suffering indicate not simply a condition curable by simple means, but serious tube and ovary mischief curable by no means short of an abdominal section. On the other hand, and I believe there is a growing feeling in the profession that conservative treatment is much more often called for, and that the endometrium is the seat of progressive mischief. About ten years ago, Dr. Gill Wylie brought to our notice his stem drain, which he introduced into the uterus after curettement, and, two years ago, Dr. William Polk emphasized that treatment by giving us his simple but very efficacious operation, and with it a report of a large and extremely interesting class of cases, in which there was considerable tubal and ovarian mischief, kept up and dependent upon endometritis. The operation consists, first, in thoroughly washing the vulva and vagina with soap and water, and then irrigating with 1 × 2000 corrosive sublimate solution. The cervix is to be freely dilated, and the cavity of the uterus thoroughly curetted, and then again freely irrigated with the solution. Polk's cervical speculum is then introduced, and the whole uterus packed with gauze. He takes thin strips of gauze, one-half an inch in width and three feet in length, and places them during the operation in a solution, 1 × 500, of corrosive sublimate, and quickly squeezes out the excess of fluid, and then places them for a few seconds in pure water, and, with the aid of a Sim's screw, packs the uterus, being careful to fill the cornuæ. This packing is left *in situ* for one week, and is then removed with a pair of long dressing forceps. If there still remains tenderness, the packing is renewed. I prefer iodoform sprinkled upon the gauze, as I found, in my first cases, that the gauze drain soon became very offensive. So favorably has this method of treatment been received in New York, that many operators first curette and pack the uterus before doing a section, so as to get a perfectly clean field, and either complete the section upon the same day or a few days later. I have done this operation eighteen times, and for a varied class of cases, and in none have I seen any fever or constitutional disturbance follow, showing positively that, with reasonable care and with the strictest antiseptic precautions, in properly selected cases, this procedure is practically without danger. And so satisfied have I been with the results, that I commend it to you very strongly. It is claimed, and I believe justly, that the great advantage of packing the uterus after curetteage is that it stimulates quicker and more perfect involution; that it promotes more perfect and permanent drainage by insuring patency of the cervical canal; that it assists in the restoration of a malplaced organ by supplying a soft support as well as drain; and that the mucous walls of the uterus, being separated by the gauze, a healthy epithelium is soon produced. I have seen the most inveterate cases of uterine leucorrhea, associated with metrorrhagia and menorrhagia and painful menstruation, cured by this simple procedure, when a long course of uterine applications of iodine, carbolic acid, etc., etc., had failed to bring about any improvement. But I have also been equally disappointed in cases of painful menstruation, where I had confidently expected a cure—in cases where no marked tube trouble was present—and which shows conclusively that we can often have dysmenorrhea, not dependent upon a hyperesthetic condition of the mucous membrane of the internal os, but an irritable nervous system, and relieved only by such means as will strengthen the natural vigor of the whole economy.

I prefer to do this operation in those cases of endometritis associated with slight tubal thickening, when heretofore I used applications of iodine, carbolic acid, and intrauterine galvanic treatments, believing that it is less dangerous to thoroughly clean the uterus at one sitting than to subject the patient to the possible dangers of increasing tubal trouble by too frequent intrauterine medication.

Of course, I do not wish to be understood that this operation is to take the place of that brilliant surgical procedure, salpingotomy and salpingo-ophorectomy; because nothing short of a laparatomy can cure a big pus tube with its accompanying ovarian abscess; but I do believe that tubal and ovarian inflammations, short of pus collections, are often cured by dilatation curettement and uterine drainage, and without adding a large element of danger either to the existing inflammations, or to the possibilities of remoter mischief.

And lastly, it must not be thought that all cases of chronic endometritis require these vigorous measures, because, in many instances, simple constitutional remedies, which have in view the bringing up of the general tone of the system and the correcting of any existing diathesis, as gout, scrofula, syphilis, etc., etc., are all that is necessary. Moreover, if there be a marked laceration of the cervix, with ectropion of its lips, an Emmet's operation alone, or with a curettement, is indicated; and, in fact, every recognized cause of the disease must be considered, to get the best results. Therefore, the physician must look farther than the mere confines of the uterus and its adjacent structures in treating its diseases, and will need to search, studiously and patiently, the whole economy, and bring to bear, in the treatment of these special diseases, the experience and judgment of a well-educated general practitioner of medicine.

78 Niagara Street.

